# Biomechanical effect of pedicle screw distribution in AIS instrumentation using a segmental translation technique: computer modeling and simulation

**DOI:** 10.1186/s13013-017-0120-4

**Published:** 2017-04-17

**Authors:** Xiaoyu Wang, A. Noelle Larson, Dennis G. Crandall, Stefan Parent, Hubert Labelle, Charles G. T. Ledonio, Carl-Eric Aubin

**Affiliations:** 1 Department of Mechanical Engineering, Polytechnique Montréal, P.O. Box 6079, Downtown Station, Montreal, Quebec H3C 3A7 Canada; 2 Sainte-Justine University Hospital Center, 3175, Cote Sainte-Catherine Road, Montreal, Quebec H3T 1C5 Canada; 3 Department of Orthopedic Surgery, Mayo Clinic, 200 1st Street SW, Rochester, MN 55905 USA; 4 Sonoran Spine Center and Research Foundation, 1255 W Rio Salado Pkwy, Suite 107, Tempe, AZ 85281 USA; 5 Department of Orthopaedic Surgery, University of Minnesota, 2450 Riverside Avenue South, Suite R200, Minneapolis, MN 55454 USA

**Keywords:** Pedicle screw, Adolescent idiopathic scoliosis, Instrumentation, Biomechanical modeling, Simulation, Screw pattern, Screw density, Screw distribution

## Abstract

**Background:**

Efforts to select the appropriate number of implants in adolescent idiopathic scoliosis (AIS) instrumentation are hampered by a lack of biomechanical studies. The objective was to biomechanically evaluate screw density at different regions in the curve for AIS correction to test the hypothesis that alternative screw patterns do not compromise anticipated correction in AIS when using a segmental translation technique.

**Methods:**

Instrumentation simulations were computationally performed for 10 AIS cases. We simulated simultaneous concave and convex segmental translation for a reference screw pattern (bilateral polyaxial pedicle screws with dorsal height adjustability at every level fused) and four alternative patterns; screws were dropped respectively on convex or concave side at alternate levels or at the periapical levels (21 to 25% fewer screws). Predicted deformity correction and screw forces were compared.

**Results:**

Final simulated Cobb angle differences with the alternative screw patterns varied between 1° to 5° (39 simulations) and 8° (1 simulation) compared to the reference maximal density screw pattern. Thoracic kyphosis and apical vertebral rotation were within 2° of the reference screw pattern. Screw forces were 76 ± 43 N, 96 ± 58 N, 90 ± 54 N, 82 ± 33 N, and 79 ± 42 N, respectively, for the reference screw pattern and screw dropouts at convex alternate levels, concave alternate levels, convex periapical levels, and concave periapical levels. Bone-screw forces for the alternative patterns were higher than the reference pattern (*p* < 0.0003). There was no statistical bone-screw force difference between convex and concave alternate dropouts and between convex and concave periapical dropouts (*p* > 0.28). Alternate dropout screw forces were higher than periapical dropouts (*p* < 0.05).

**Conclusions:**

Using a simultaneous segmental translation technique, deformity correction can be achieved with 23% fewer screws than maximal density screw pattern, but resulted in 25% higher bone-screw forces. Screw dropouts could be either on the convex side or on the concave side at alternate levels or at periapical levels. Periapical screw dropouts may more likely result in lower bone-screw force increase than alternate level screw dropouts.

## Background

Pedicle screw fixation has become the state-of-the-art instrumentation for the surgical treatment of adolescent idiopathic scoliosis (AIS), resulting in better deformity correction and lower revision surgery rates compared to hybrid or hook-rod constructs [1–3]. However, wide variation in clinical practice persists regarding the number and distribution of pedicle screws used, as well as the surgical techniques for the treatment of pediatric scoliosis.

Screw density is defined as the number of screws per level fused. There may be multiple clinical and biomechanical factors in determining screw density and distribution. Certain screw types and distributions are required in order to perform specific correction maneuvers, such as apical vertebral derotation and segmental vertebral derotation [4]. The effect of screw density depends also on the construct design. Some screw types and patterns tended to overconstrain the instrumented spine generating high (overconstraining) bone-screw forces in high-density screw constructs, such as monoaxial screws [5]; screws with multiple degrees of adjustability allowed the overconstraining effect to be reduced and segmental translation to be performed in a gradual and incremental way to lower the overall bone-screw force level [5, 6].

A structured literature review revealed that the mean reported screw density varies from 1.04 to 2.0, whereas the average curve corrections only varied from 64 to 70% [7]. Some surgeons routinely use two screws at every level fused where other surgeons may use up to 46% fewer screws [8, 9]. High screw density constructs have been associated with increased operative time, blood loss, radiation exposure, instrumentation costs, and risk of screw-related complications [10–14]. Constructs with fewer screws may have benefits for optimal use of health care resources [7, 8]. Some studies note improved percent correction of major coronal curve in the high screw density cohort [8, 9]; but, in other studies, no significant difference in outcome was found between the high and low screw density groups [10, 15, 16].

Until recently, studies of screw density have been underpowered, included hybrid constructs, or based on retrospective review of clinical data. Further, there is a lack of biomechanical data guiding screw number and placement. Thus, practice is mostly based on individual preferred technique, and scientific progress is limited by the inability to test alternative screw patterns on a given patient. In contrast to studies based on clinical data analysis, biomechanical studies using computerized patient-specific models allow the assessment and comparison of variable screw numbers and patterns with different correction techniques simulated for the same case. The objective of this study was to use computerized patient-specific spine models to biomechanically evaluate screw dropouts at different regions in the curve for AIS correction to test the hypothesis that alternative screw patterns do not compromise anticipated correction in AIS when using segmental translation as the primary correction technique.

## Methods

Numerical simulations of posterior spinal instrumentations were performed using computerized patient-specific biomechanical models of 10 AIS patients in order to assess the effect of screw density on curve correction and bone-screw forces. With the institutional review board approval, the cases were randomly selected from AIS patients having undergone instrumented spinal fusion at our university hospital center during the last 6 years. Clinical indices are provided in Table 1. Modeling and simulation details are presented in the following subsections.

**Table 1:** Clinical indices

Case no.		1	2	3	4	5	6	7	8	9	10
Sex		F	F	F	F	F	M	F	F	F	F
Age		14	16	19	17	14	15	16	15	15	14
Height (cm)		154	162	162	168	170	172	165	170	159	148
Weight (kg)		52	56	47	56	59	55	53	48	59	39
Lenke classification		1A	1A	3B	4A	3B	3C	1A	1A	3C	2A
MT superior end vertebra		T6	T6	T5	T5	T5	T7	T5	T4	T6	T5
Apical vertebra		T9	T11	T8	T8	T8	T10	T9	T8	T9	T9
Inferior end vertebra		T12	L2	T11	T11	T12	L1	L1	T12	T12	T11
PT Cobb	Preop.	32°	31°	39°	52°	31°	34°	28°	9°	40°	42°
	Left bending	22°	10°	12°	17°	24°	6°	20°	5°	15°	28°
	Right bending	35°	41°	42°	54°	35°	37°	32°	20°	41°	46°
MT Cobb	Preop.	55°	52°	58°	60°	64°	62°	44°	48°	67°	51°
	Left bending	59°	60°	63°	64°	65°	62°	73°	55°	63°	60°
	Right bending	29°	25°	29°	38°	50°	27°	10°	30°	35°	30°
MT AVR	Preop.	16°	17°	18°	19°	19°	20°	19°	18°	22°	19°
TL/L Cobb	Preop.	37°	37°	39°	30°	42°	48°	35°	39°	40°	32°
	Left bending	2°	5°	20°	9°	5°	30°	10°	10°	9°	2°
	Right bending	49°	50°	42°	40°	65°	50°	42°	45°	70°	49°
TL/L AVR	Preop.	5°	3°	7°	7°	3°	11°	6°	11°	9°	3°
Kyphosis	Preop.	7°	23°	37°	28°	22°	11°	20°	18°	7°	15°
Lordosis	Preop.	42°	37°	27°	45°	33°	15°	47°	40°	30°	32°
UIV		T4	T4	T4	T3	T3	T4	T4	T4	T3	T3
LIV		L2	L3	L1	L1	L1	L2	L2	L2	L2	L1

*F* female, *M* male, *PT* proximal thoracic, *MT* main thoracic, *TL/L* thoracolumbar/lumbar, *AVR* apical vertebral rotation, *UIV* upper instrumented vertebra, *LIV* lower instrumented vertebra

### Computerized patient-specific biomechanical spine model

Three-dimension (3D) spine geometry of the selected cases was built using calibrated preoperative coronal and lateral radiographs and 3D multi-view reconstruction techniques [17]. The process began with the identification of key anatomical landmarks of each vertebra, typically, the pedicles, vertebral endplate middle and corner points, and transverse and spinous process extremities. The 2D coordinates of these landmarks allowed the determination of their 3D coordinates in space, which was done using a self-calibration and optimization algorithm [17, 18]. The reconstruction process was completed by registering detailed vertebral models using the 3D coordinates of the key landmarks and a free form deformation technique [17, 18]. Average accuracies for pedicles and vertebral bodies were 1.6 mm (SD 1.1 mm) and 1.2 mm (SD 0.8 mm), respectively [18].

**Fig. 1:**
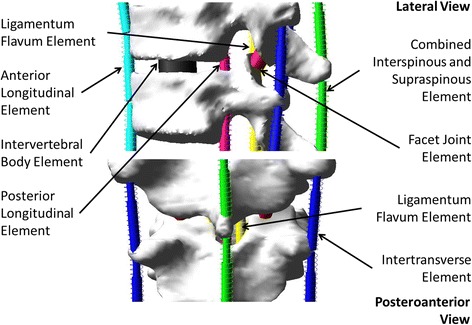
Multibody modeling elements of a functional spinal unit

Vertebrae from T1 through L5 and the pelvis were modeled as rigid parts which were connected with multiple flexible elements respectively representing the biomechanical effect of the intervertebral disc, anterior longitudinal ligament (ALL), posterior longitudinal ligament (PLL), ligamentum flavum (LF), intertransverse ligament (ITL), facet joint capsule (FC), and interspinous ligament (ISL) combined with supraspinous ligament (SSL). Six translational springs were used to respectively represent (1) ALL, (2) PLL, (3) LF, (4) left ITL, (5) right ITL, and (6) the combined effect of ISL and SSL. The biomechanical behavior of the facet joints is more complex compared to the other intervertebral ligamentous elements; they were respectively represented with a six-dimensional general spring [19]. A primary general spring was used to represent the intervertebral disc to which the effect of all elements and factors which were not explicitly modeled in this study was incorporated by introducing weighting factors to the diagonal elements of its stiffness matrix, e.g., the rib cage increased the stiffness of the thoracic spine by 40, 35, and 31% respectively in flexion/extension, lateral bending, and axial rotation [20]. The multibody modeling elements are illustrated in Fig. 1.

The six translational springs were modeled as cable-like elements on the computer-aided engineering platform, Adams/View, Version MD Adams 2010 (MSC Software Corporation, Santa Ana, CA, USA). Their stiffness in compression were set to null and those in traction were adapted to the reported experiment results on cadaveric specimens, e.g., 23.6 N/mm (ALL), 24.9 N/mm (PLL), 32.6 N/mm (LF), 12.9 N/mm (ITL), and 32.1 N/mm (ISL combined with SSL) [21–23] for T6-T7 functional spinal unit. Instrumented posterior spinal fusion with pedicle screw fixation involves the removal of the facet joint capsules and the spinous process. The biomechanical effects of pedicle screw placement surgical procedure were modeled by removing the facet joint capsule and the interspinous model elements, whose mechanical properties were calibrated using experiment data reported in the literature, i.e., osteotomies involved in pedicle screw placement procedure reduced the stiffness of a functional spine unit by 17% in axial rotation [24–27], 15% sagittal plane flexion [24, 28, 29], 3.8% in coronal plane bending [24], and 14% in axial compressive load [30–33]. The stiffness matrix of the primary general spring was calibrated such that the load-displacement simulations with the model of a functional spinal unit reproduced the reported load-displacement data [34–37]. All model element stiffness were further adjusted such that side bending simulations reproduced the Cobb angles measured on the patient’s side bending radiographs using a similar optimization technique reported in [38, 39].

### Biomechanical modeling and simulations of spinal instrumentation

**Fig. 2:**
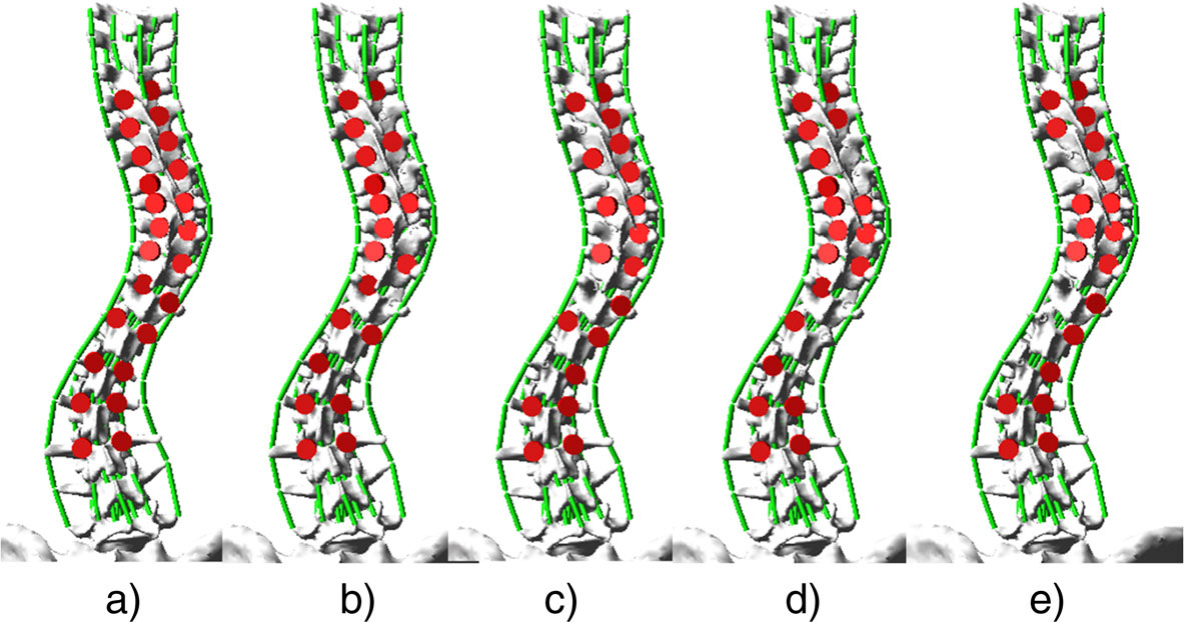
**a** Reference screw pattern (bilateral screws at every level fused). **b** Screw pattern with convex alternate screw dropouts. **c** Concave alternate screw dropouts. **d** Convex periapical screw dropouts. **e** Concave periapical screw dropouts

**Fig. 3:**
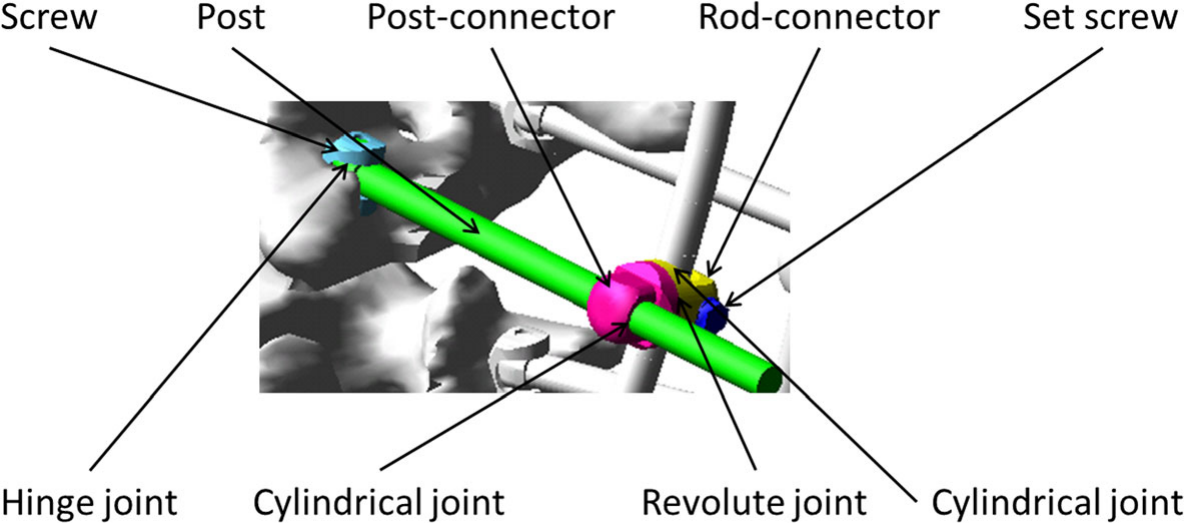
Polyaxial pedicle screw with dorsal height adjustability

Based on the actual instrumented fusion levels of the selected cases and the well-accepted alternative screw densities [4], five screw patterns were biomechanically evaluated on each case. The screw patterns were a reference screw pattern with bilateral screws at every level fused and four alternative patterns with mean 23% fewer screws (21 to 25%). Screw dropouts in the alternative patterns were respectively on the convex and concave sides and at alternate levels or periapical levels (Fig. 2). The modeled bone-screw connection and correction technique was based on polyaxial pedicle screws with dorsal height adjustability (4.5–5.5 mm diameters for the thoracic spine and 5.5–6.0 mm diameters for the lumbar spine) (Fig. 3) [6]. The screw kinematic design allows the translation of each pedicle screw toward the rod from any distance and at any angle, with the ability to rigidly lock the construct at any point between partial and complete corrections [6]. The simulated correction technique was simultaneous two-rod segmental translation. The biomechanical model of the rods was based on 5.5 mm Cobalt-chrome rods. The contouring angle of the convex rod was 25° as measured over the thoracic spinal segment, and the contouring angle of the concave rod was 35°. Modeling of instrumentation constructs, simultaneous two-rod segmental translation and the boundary conditions have been realized and validated in a previous study [6].

## Results

The computed geometric indices from the reconstructed preoperative spine models and the simulated instrumented spine models are presented in Table 2. In the 40 simulations with the alternative screw patterns, the final simulated Cobb angle differences varied between 1° to 5° in 39 simulations and 8° in 1 simulation compared to the reference maximal density screw pattern. Thoracic kyphosis and apical vertebral rotation were within 2° of the reference screw pattern.

**Table 2:** Computed geometric indices from the reconstructed preoperative spine models and the simulated instrumented spine models

Case no.	1	2	3	4	5	6	7	8	9	10
Main thoracic Cobb angles										
Preop.	55°	52°	58°	60°	64°	62°	44°	48°	67°	51°
Pattern no. 1 (reference)	18°	10°	18°	14°	24°	21°	17°	15°	28°	17°
Pattern no. 2	18°	12°	22°	15°	25°	21°	17°	16°	29°	17°
Pattern no. 3	18°	10°	17°	22°	24°	21°	15°	16°	29°	17°
Pattern no. 4	16°	12°	20°	14°	24°	22°	16°	15°	30°	17°
Pattern no. 5	21°	9°	19°	19°	24°	21°	16°	15°	30°	17°
Thoracic kyphosis										
Preop.	7°	23°	37°	28°	22°	11°	20°	18°	7°	15°
Pattern no. 1 (reference)	27°	31°	33°	22°	24°	28°	29°	27°	24°	25°
Pattern no. 2	26°	30°	33°	20°	24°	28°	29°	27°	24°	25°
Pattern no. 3	27°	32°	33°	21°	23°	28°	29°	27°	24°	25°
Pattern no. 4	28°	31°	32°	22°	23°	28°	29°	27°	24°	25°
Pattern no. 5	26°	31°	33°	21°	24°	29°	29°	27°	24°	25°
Main thoracic apical vertebral rotation										
Preop.	16°	17°	18°	19°	19°	20°	19°	18°	22°	19°
Pattern no. 1 (reference)	15°	18°	18°	17°	17°	19°	18°	17°	20°	18°
Pattern no. 2	14°	18°	17°	16°	17°	19°	19°	17°	21°	19°
Pattern no. 3	15°	18°	18°	15°	17°	19°	17°	17°	20°	18°
Pattern no. 4	15°	18°	18°	17°	16°	19°	18°	17°	21°	19°
Pattern no. 5	14°	18°	19°	15°	17°	19°	17°	17°	20°	18°

Pattern no. 1 (reference): bilateral screws at every level fused; pattern no. 2: convex alternate screw dropouts; pattern no. 3: concave alternate screw dropouts; pattern no. 4: convex periapical screw dropouts; pattern no. 5: concave periapical screw dropouts

**Fig. 4:**
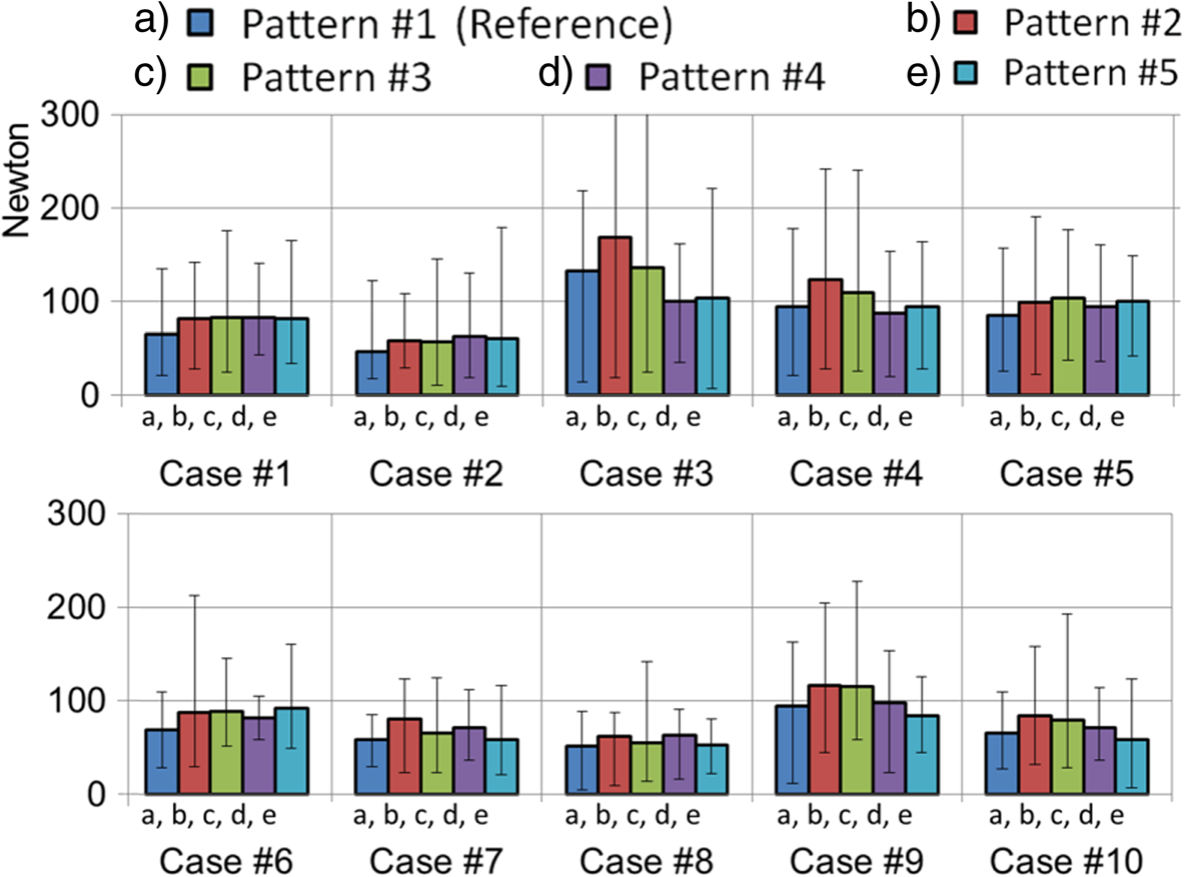
Average (*bars* = min, max) bone-screw forces (pattern no. 1 (reference): bilateral screws at every level fused; pattern no. 2: convex alternate screw dropouts; pattern no. 3: concave alternate screw dropouts; pattern no. 4: convex periapical screw dropouts; pattern no. 5: concave periapical screw dropouts)

**Fig. 5:**
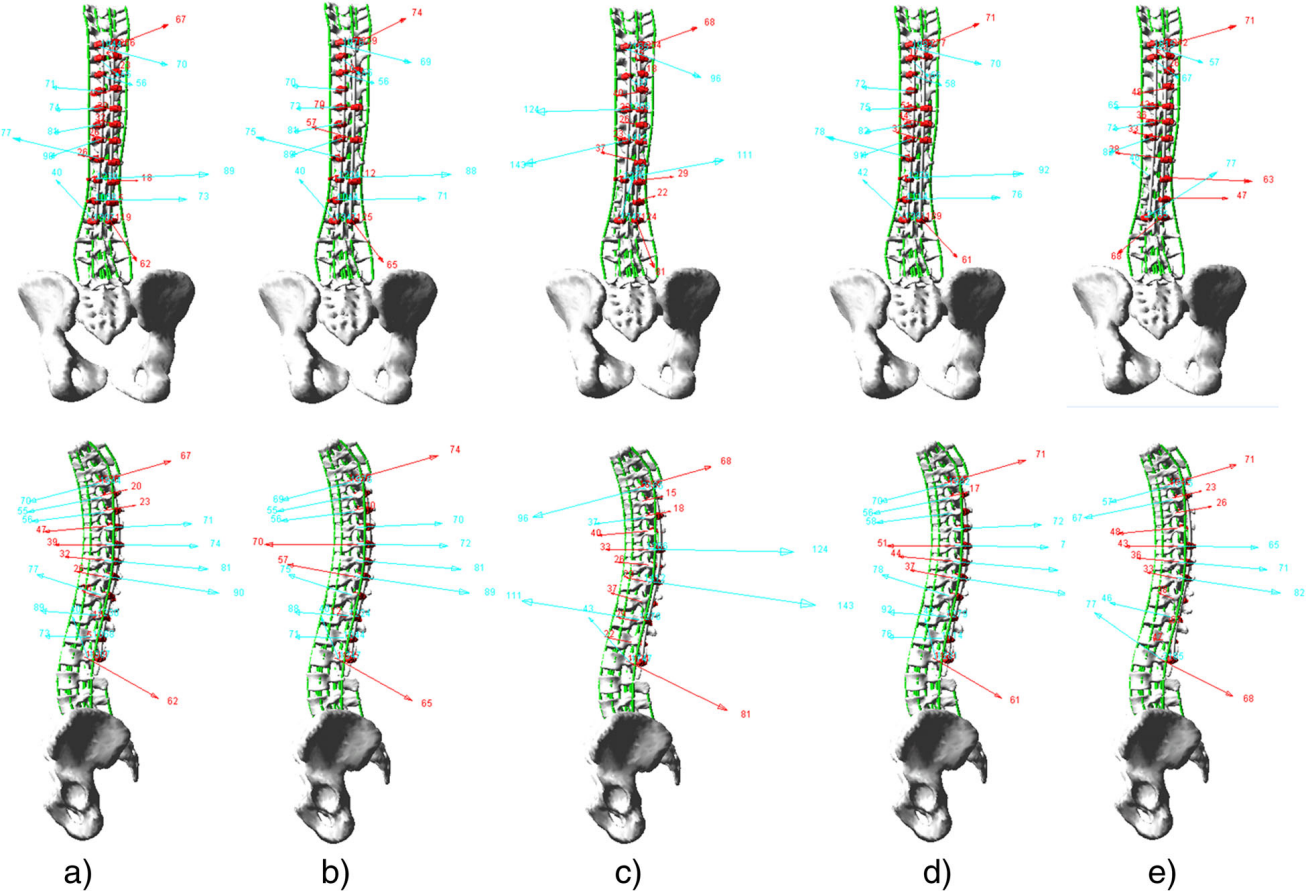
Sample bone-screw force patterns (case no. 8) with (**a**) the reference screw pattern (bilateral screws at every level fused) and screw patterns with (**b**) convex alternate screw dropouts, (**c**) concave alternate screw dropouts, (**d**) convex periapical screw dropouts, and (**e**) concave periapical screw dropouts (*red arrows*: convex side bone-screw force vectors; *cyan arrows*: concave side bone-screw force vectors)

Average bone-screw force was computed for each simulation and the results are presented in Fig. 4. The overall bone-screw force was 76 ± 43 N (5–219 N) for the maximal density reference screw pattern. They were 96 ± 58 N (10–468 N), 90 ± 54 N (11–353 N), 82 ± 33 N (17–162 N), and 79 ± 42 N (7–222 N), respectively, for the four alternative screw patterns with screw dropouts at convex alternate levels, concave alternate levels, convex periapical levels, and concave periapical levels, which were respectively 26, 17, 7, and 4% higher than the reference screw pattern. Bone-screw forces for the alternative patterns were statistically higher than the reference pattern (*p* < 0.0003). Alternate dropout screw forces were higher than periapical dropouts (*p* < 0.05). There was no statistical bone-screw force difference between convex and concave alternate dropouts (*p* > 0.28). Although there was no statistical bone-screw force difference between convex and concave periapical dropouts (*p* > 0.25), the convex periapical screw dropouts had less impact on bone-screw force vector pattern in some of the cases in both the coronal and the sagittal plane, i.e., bone-screw force vector pattern was the closest to the reference screw pattern. Bone-screw force vectors for a representative case are provided in Fig. 5.

## Discussion

Comparing alternative screw patterns respectively with the maximal density reference screw pattern, differences in the final simulated MT Cobb angles, thoracic kyphosis, and apical vertebral rotation did not exceed 5° for all except one case. These differences are within the accepted systematic error found in clinical Cobb angle measurements [40]. The mean correction of each of the alternative screw pattern was within 2° of the mean correction of the reference screw pattern. Based on a previous study on 279 AIS patients [9] and a structured literature review on AIS instrumentations [7], the population mean of major curve Cobb angles was estimated to be 55° and the population mean of percent corrections of major curves was estimated to be 67% with a standard deviation of 14%. The correction difference to be detected was set to 5° (11% difference in percent correction). There was no statistical difference between the reference screw pattern and the alternative screw patterns in terms of percent corrections of major curves, with 5% of type I error and a statistical power of 70%.

**Fig. 6:**
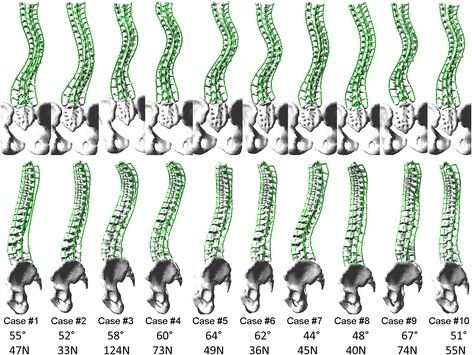
Posteroanterior and lateral views of the preoperative spine models, major curve Cobb angles and the average bone-screw forces with bilateral screws at each level fused

Alternative screw patterns with fewer screws resulted in higher overall bone-screw forces. Previous studies showed that higher density screw patterns had usually higher bone-screw forces due to the overconstraining effect [41]. The difference can be attributed to differences between the construct designs, type of screws, and simulated correction techniques. Higher bone-screw forces were generally associated with higher Cobb angles when percent corrections were similar; bone-screw forces in curves of higher Cobb angles may be more sensitive to screw density and distribution. The number of vertebrae in the major curve and the local shape of the curve seem to have an important effect on the average bone-screw force; higher forces were more seen in cases in which the major curves spanned fewer vertebrae and were more angular (Fig. 6). In other words, curves which span longer spinal segment and whose curvature does not varies significantly tend to have lower bone-screw forces. A sharp, short angular curve with a high local Cobb angle requires greater corrective forces to align the spine to the smoothly contoured spinal rods. Short, angular curves may therefore be more sensitive to screw density and distribution, which should be taken into consideration in addition to curve flexibility. Significantly higher bone-screw forces in some cases may be explained by the fact that the curve spanned a shorter spinal segment and was more angular (cases 3, 4, and 9). In the sagittal plane, the spinal profiles in some cases may have a better match with the rod shapes than in other cases, which should also have an impact on the final bone-screw forces. Screw density and distribution should therefore be determined by taking into account the local geometric characteristics of the curve in both the sagittal and coronal planes in addition to the curve type, deformity magnitude, and spinal stiffness.

In summary, with simultaneous two-rod segmental translation using polyaxial pedicle screws with dorsal height adjustability, there was no statistical difference between convex side screw dropouts and concave side screw dropouts. Periapical screw dropouts may more likely result in lower bone-screw force increase than alternate level screw dropouts. The number of vertebrae spanned by the major curve or the sharpness of the curve affects the bone-screw forces; screw dropouts should be reduced within short structural curves with high local curvature. Screw density and distribution should be determined by taking into account the local geometric characteristics of the curve in both the sagittal and coronal planes and the shape of the rods in addition to the curve type, deformity magnitude, and spinal stiffness.

Different correction techniques and instrumentation construct designs may have important roles in curve correction and bone-screw forces. Since the simulated correction technique was simultaneous two-rod segmental translation using polyaxial pedicle screws with dorsal height adjustability, findings in this study may not be directly applied to other techniques, types of screws, and construct designs. However, based on the fundamental laws of mechanics, for an equivalent curve correction, the overall effective corrective forces should be at an equivalent level independent of correction techniques and construct designs. Findings in this study provided therefore useful data on the overall effect of screw density and distribution. Knowledge of potential bone-screw forces in AIS instrumentation using alternative computer-simulated screw constructs can help surgeons select the best possible screw configuration specific for the patient. All bone-screw forces may not contribute to the actual curve correction due to the high mechanic complexity of the instrumented spine. Parts of the bone-screw forces are the “true corrective forces,” which are necessary and sufficient to achieve the desired correction and the rest are overconstraining forces which are induced when forcing to ensure proper rod seating and locking at all pedicle screws as required by the construct design [42]. The effects of screw design, and density and distribution on true corrective forces and overconstraining forces need to be investigated with more AIS cases using various correction techniques and construct designs. Depending on the pedicle size of each individual patient, the pedicle screw diameter used varies among patients, which should be an important factor in determining the screw density and distribution and needs to be investigated.

This study is limited by the available experimental data to calibrate and describe the biomechanical properties of the scoliotic spine model. However, the modeling technique has been adapted to make the best of the available calibration data to meet the needs of this study. Some simplifications were made, such as modeling the vertebral bodies as rigid parts, limiting the model solving in the quasistatic domain, and approximating the intervertebral connection with limited number of elastic elements. Since the focus was on the overall comparative curve correction and bone-screw forces, these simplifications and approximations were considered as adequate for this study. To establish baseline data for screw density and distribution, studies through simulations using computerized biomechanical models should be combined with prospective clinical studies and biomechanical experiments. The computerized model will be refined and better calibrated using the more comprehensive clinical and experimental results and then used to perform more extensive studies which may not be possible in a clinical and experimental context.

## Conclusions

Deformity correction can be achieved with 23% fewer screws than maximal density screw pattern, in which screw dropouts could be either on the convex side or on the concave side at alternate levels or at periapical levels. Using fewer screws resulted in higher average bone-screw forces. Findings in this study provided preliminary data on the effect of screw density and distribution. Further studies should be conducted on more screw densities and distributions using different correction techniques and different instrumentation in order to acquire comprehensive biomechanical knowledge to assist in individualized surgical treatment for AIS.

## Abbreviations

3D: Three-dimension
AIS: Adolescent idiopathic scoliosis
ALL: Anterior longitudinal ligament
AVR: Apical vertebral rotation
FC: Facet joint capsule
ISL: Interspinous ligament
ITL: Intertransverse ligament
LF: Ligamentum flavum
LIV: Lower instrumented vertebra
MT: Main thoracic
PLL: Posterior longitudinal ligament
PT: Proximal thoracic
SD: Standard deviation
SSL: Supraspinous ligament
TL/L: Thoracolumbar/lumbar
UIV: Upper instrumented vertebra
